# Supplementary figures and images for: Cyclone: an accessible pipeline to analyze, evaluate, and optimize multiparametric cytometry data
Source: Front Immunol. 2023 Sep 4;14:1167241. doi: 10.3389/fimmu.2023.1167241 (PMC10507399; doi:10.3389/fimmu.2023.1167241)

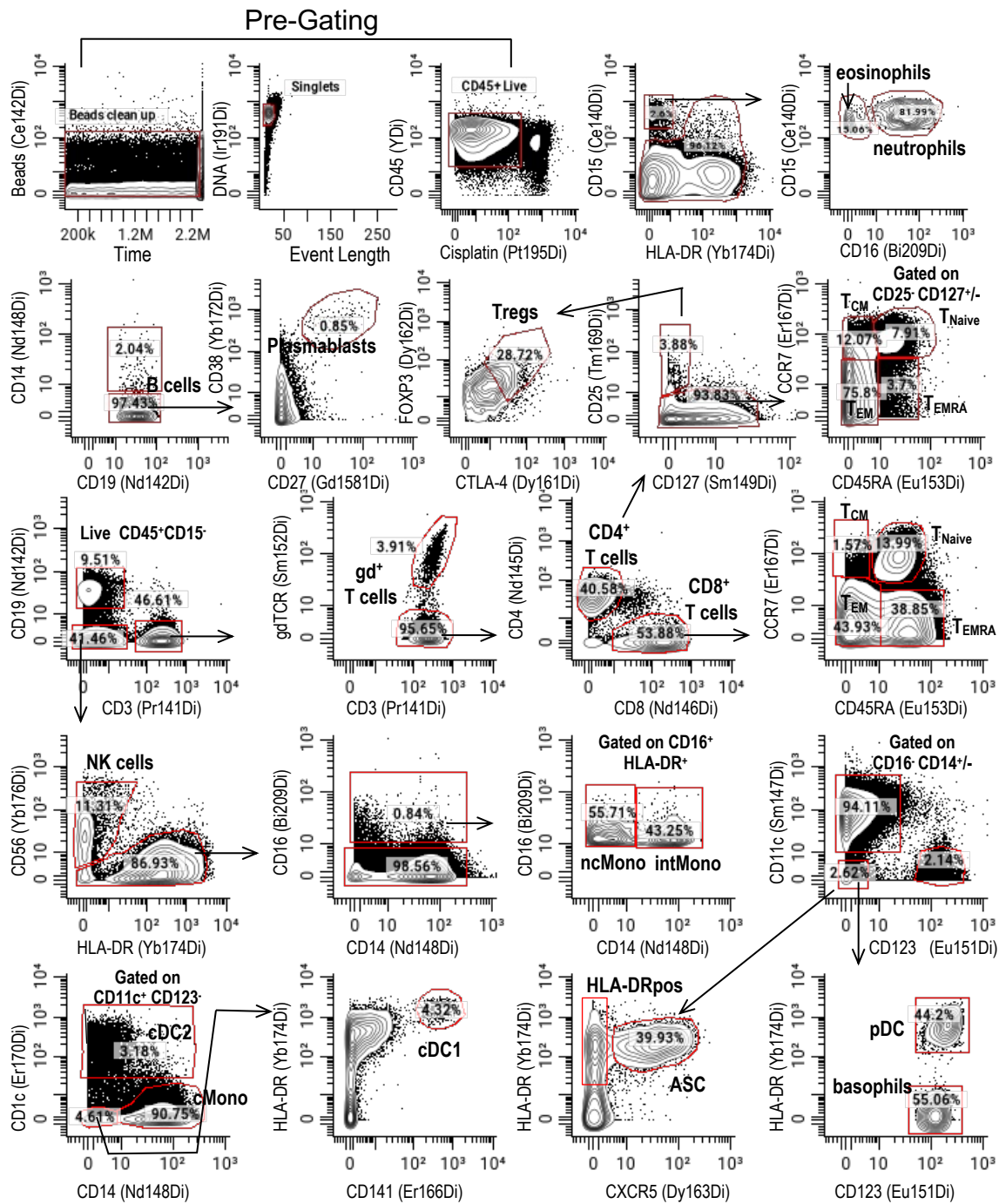

Figure Supplementary 1

Supplement: Supplementary Figure 1 — CyTOF manual gating of human PBMC. (A) Pre-Gating: gating out beads, debris, dead cells, RBC and granulocytes. (B) Hierarchical gating was applied to identify 22 “landmark” immune populations: CD14+ CD16- classical monocytes, CD14-CD16+ nonclassical monocytes, CD14+ CD16+ intermediate monocytes, cDC1, cDC2, pDC, basophils, Natural Killer cells, regulatory CD4+ T cells, CD4+ T cells (Naive, TCM, TEM, TEMRA), CD8+ T cells (Naive, TCM, TEM, TEMRA), γδ+ T cells, B cells, ASC (antibody producing cells) HLA-DRpos (CXCR5- B cells), plasmablasts. Also shown gating of eosinophils (CD15+ CD16+ HLA-DR-) and neutrophils (CD15+ CD16- HLA-DR-). [file Image_1.pdf]

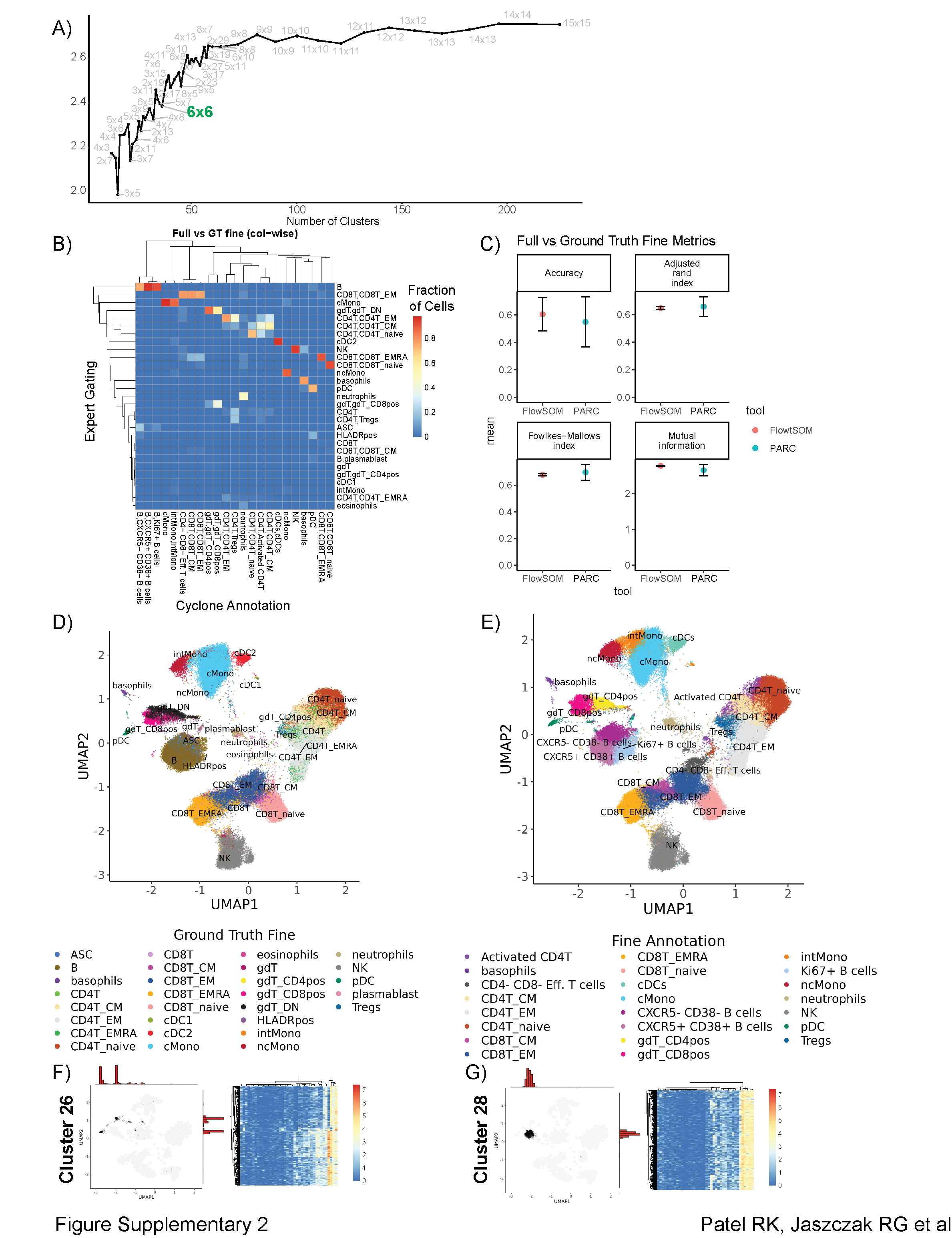

Supplement: Supplementary Figure 2 — Assessment of “Fine”-level annotations and metrics for evaluating cyclone outputs. (A) Full Davis-Bouldin index plot showing up to 200 potential clusters identified through FlowSOM. (B) Heatmap of full dataset “fine”-level annotations identifying cell types and cell subtypes, based on ground truth (GT) manual gating (rows) compared to annotated FlowSOM clusters (columns). (C) Comparison metrics based on “fine”-level annotations from two individuals. Various performance metrics were used to assess the accuracy of clusters called in the FlowSOM clustering compared to ground truth. (D) Ground Truth expert cluster “fine”-level annotation identifying broad cell types and specific cell subtypes based on manual gating. (E) FlowSOM clustering “fine”-level annotations based on CyTOF panel expression. F) Depiction of a cluster dispersed across UMAP space (Cluster 26) with a heterogenous protein expression profile compared to G) a cluster with uniform protein expression and tight UMAP localization (Cluster 28). [file Image_2.tif]

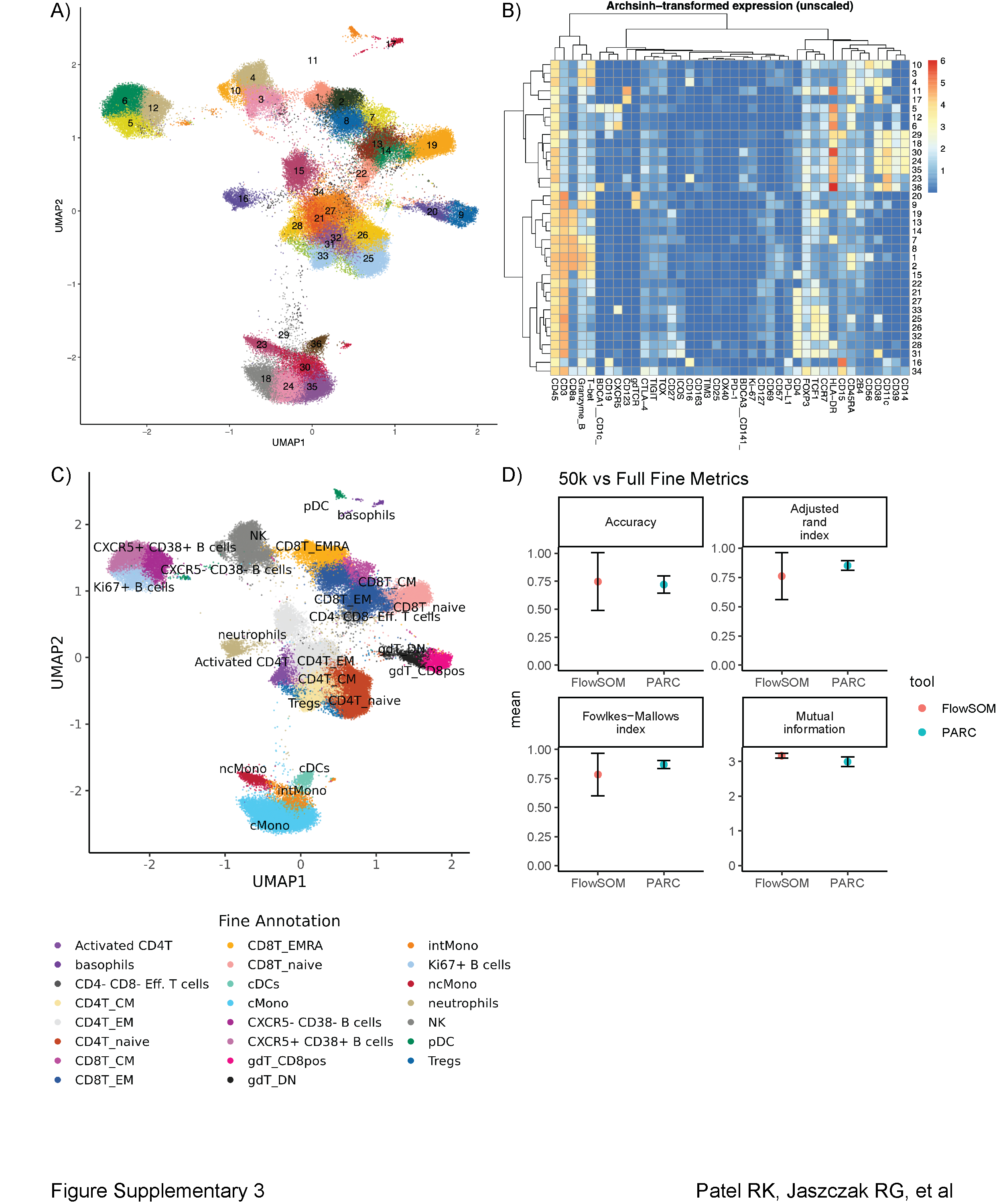

Supplement: Supplementary Figure 3 — “Fine”-level annotations after running Cyclone on the downsampled dataset. The dataset was down-sampled to 50k cells per sample and then run through cyclone. Clusters’ cell type identities were inferred by experts using Cyclone plot outputs. (A) UMAP annotated by cluster number. (B) Heatmap of median archsinh transformed expression (unscaled) per cluster, used to annotate clusters. (C) UMAP from 50k down-sample run, colored by fine annotations. (D) Comparison of per-cell annotations between the 50k down-sample versus the full dataset. Various performance metrics were used to assess the accuracy of clusters called in the downsampled dataset compared to the full dataset. [file Image_3.tif]

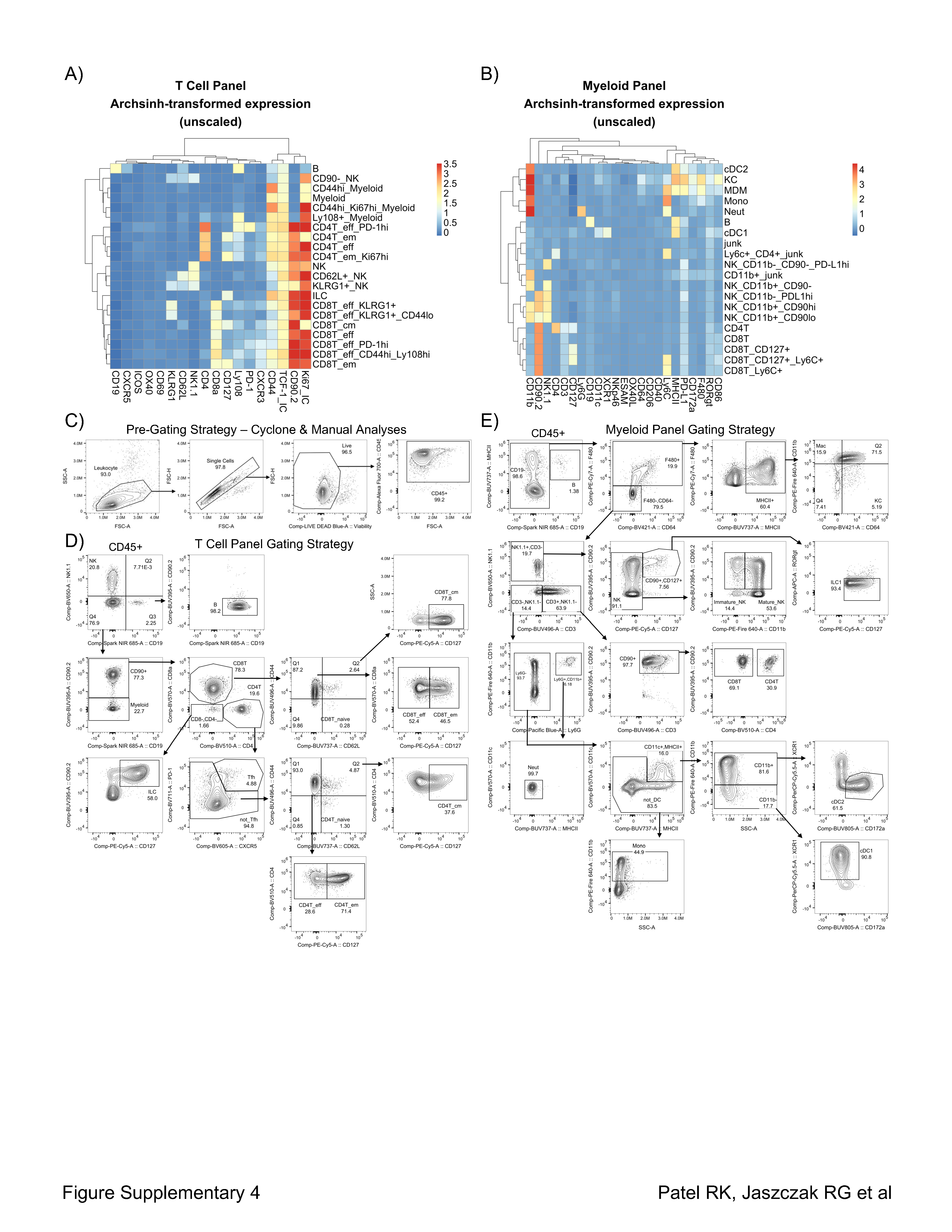

Supplement: Supplementary Figure 4 — Spectral flow cytometry cell type identification. (A) Heatmap of markers used for UMAP generation, clustering, and identification for “fine”-level Cyclone clusters for the spectral flow cytometry dataset with a T cell-focused panel presented in Figure 5A . (B) Heatmap of markers used for UMAP generation, clustering, and identification for “fine”-level Cyclone clusters for the spectral flow cytometry dataset with a Myeloid-focused panel presented in 5B . (C) Representative two-dimensional flow plots demonstrating pre-gating on live CD45+ cells before analysis with either Cyclone or expert manual gating in FlowJo. (D) Manual gating strategy for samples in the T cell-focused panel. (E) Manual gating strategy for samples in the Myeloid-focused panel. [file Image_4.tif]
